# 4DRoot: Root phenotyping software for temporal 3D scans by X-ray computed tomography

**DOI:** 10.3389/fpls.2022.986856

**Published:** 2022-09-23

**Authors:** Monica Herrero-Huerta, Pasi Raumonen, Diego Gonzalez-Aguilera

**Affiliations:** ^1^Department of Cartographic and Land Engineering, Higher Polytechnic School of Ávila, Universidad de Salamanca, Ávila, Spain; ^2^Department of Computing Sciences, Tampere University, Tampere, Finland

**Keywords:** root phenotyping, 3D modeling, X-ray computed tomography, imaging, proximal sensing

## Abstract

Currently, plant phenomics is considered the key to reducing the genotype-to-phenotype knowledge gap in plant breeding. In this context, breakthrough imaging technologies have demonstrated high accuracy and reliability. The X-ray computed tomography (CT) technology can noninvasively scan roots in 3D; however, it is urgently required to implement high-throughput phenotyping procedures and analyses to increase the amount of data to measure more complex root phenotypic traits. We have developed a spatial-temporal root architectural modeling software tool based on 4D data from temporal X-ray CT scans. Through a cylinder fitting, we automatically extract significant root architectural traits, distribution, and hierarchy. The open-source software tool is named 4DRoot and implemented in MATLAB. The source code is freely available at https://github.com/TIDOP-USAL/4DRoot. In this research, 3D root scans from the black walnut tree were analyzed, a punctual scan for the spatial study and a weekly time-slot series for the temporal one. 4DRoot provides breeders and root biologists an objective and useful tool to quantify carbon sequestration throw trait extraction. In addition, 4DRoot could help plant breeders to improve plants to meet the food, fuel, and fiber demands in the future, in order to increase crop yield while reducing farming inputs.

## Introduction

Major global challenges such as climate change, environmental degradation, and food insecurity demand cost-effective phenotyping methods to guarantee the fiber, fuel, and food necessities. Recently, image-based phenotyping has become an integral part of plant science analysis, noninvasively providing large volumes of data specifying plant architecture (Mairhofer et al., 2016; Gerth et al., 2021; Meline et al., 2021). Still, innovative digital approaches that may potentially increase the usability of breakthrough imaging technologies to potentially overcome the above challenges are urgently needed (McGrail et al., 2020).

Roots establish the connection between plants and the soil environment, being not only the critical piece to water and nutrient extraction but also assisting in carbon sequestration. Deeper rooting crops enhance soil organic carbon sequestration from the atmosphere, helping to reduce climate change and improving soil organic fertility. Moreover, deeper roots are effective against drought as they increase nitrogen capture to reduce fertilizer inputs and improve water uptake (Liu et al., 2021). In addition, variation in root system architecture (RSA) can have profoundly different effects on plant health and productivity in different environments (Lynch, 1995; York et al., 2013). Thus, accurate quantification of root traits helps breeders select favorable root characteristics regarding not only carbon farming and crop production but also soil degradation. However, our understanding of RSA has been hindered by its complex three-dimensional branching topology (Morris et al., 2017; Dowd et al., 2021; Shao et al., 2021). Recent improvements in image-based technologies such as X-ray computed tomography (CT) and magnetic resonance imaging (MRI) provide a 3D model of the root. They are commonly used to phenotype roots in lab conditions inside pots packed with soil and substrates (van Dusschoten et al., 2016; Atkinson et al., 2019; Takahashi and Pradal, 2021; Teramoto and Uga, 2022).

Currently, there are several software solutions based on 2D image analysis for scanned roots, such as the commercial WinRHIZO or the open-source RhizoVision Explorer. These solutions provide an easy-to-use interface, fast image processing, and reliable measurements such as length, diameter, area, and volume. WinRHIZO is a closed-source software released in 1993 (Arsenault et al., 1995) based on the principle of standardizing the use of desktop scanners and image analysis algorithms. Being unable to predict root order (i.e., the topological branching structure), RhizoVision Explorer (Seethepalli et al., 2021) improves the accuracy of the volumetric measurements and adds other traits such as angles, root depth, and convex hull. Another approach is using image solutions from conventional cameras. DynamicRoot (Symonova et al., 2015) is based on temporal voxelized reconstructions using multi-view imaging. A full branching hierarchy and traits such as volume, length, number, diameter, tortuosity, and angle are computed. As downsides, the results are affected by the topological errors in the segmentation (disconnected and loop root components) and a time series is required to compute the correct hierarchy. Newly, DIRT/3D (Liu et al., 2021) was proposed as an image-based 3D root phenotyping system by structure from motion (SfM) and the following computational analysis. DIRT/3D measures architecture traits (e.g., whorl distances, number, angles, and diameters of both root ball and brace roots) from mature field-grown maize root systems. The methodology to compute the traits is based on transversal top-down sections of the root point cloud. Another software solution for root phenotyping by XCT data is TopoRoot (Zeng et al., 2021) that uses a stack of 2D image slices from mature maize root systems. It is based on computer graphics algorithms such as topological simplification and 3D skeletonization. Traits such as number, length, thickness, angle, tortuosity (waviness of the growth pattern), and hierarchy are obtained.

All the above-cited solutions require washing the roots before being scanned, thereby losing the spatial context except DynamicRoot, which uses a gel medium to grow roots in unrealistic conditions. To solve this issue, approaches to automatically segment roots from the soil by X-ray CT systems inside pots were advanced. This is the case for the Rootine (Gao et al., 2019) procedure that improves the detection of fine roots; however, it only calculates the root length by medial axis-based skeletonization processes as a phenotypic trait. As an improvement, RootForce (Gerth et al., 2021) based on the Rootine procedure extracts more traits such as root volume and root growth angles by Reeb graph-based skeletonization (Biasotti et al., 2008; Ge et al., 2011). Moreover, there is a second version of Rootine capable of not only assessing root length but also integrating root diameter analyses (Phalempin et al., 2021).

A completed review of the existing computational approaches for root system tracking by 3D X-ray CT data is done in Xu et al. (2018). Some software tools are highlighted but they are not able to automatically compute root traits. However, to the best of our knowledge, no software tool exists to parameterize the roots by 3D geometric primitives that add the 3D characterization by listing RSA traits. Therefore, the plant science community urgently requires advanced approaches in the volumetric characterization of RSA. As a result, this article presents a root phenotyping software for 3D scans that not only extracts values of significant root traits but also records topological and hierarchical branching structure to quantitatively assist 3D dynamics and RSA description. Moreover, 4DRoot has the ability to analyze the time series of 3D CT scans to evaluate the spatial and temporal dynamics of roots. The entire approach is optimized to accurately, automatically, and robustly quantify traits, allowing high-throughput root phenotyping using X-ray CT scans.

## The 4DRoot software tool

### Implementation

The code is based on TreeQSM^1^ (Raumonen et al., 2013) but applied to X-ray CT root scans as a 3D surface geometry. We adapted the code to fit 3D scans from roots into flexible cylindrical quantitative structure models (QSM), following topological, hierarchical, and geometric rules. A QSM of a root is a model of the root structure that describes quantitatively its basic topological (root structure), geometric, and volumetric properties. These include properties such as the number of ramifications in total and in any ramification order, the parent–child relations of the ramifications and lengths, volumes, and angles of individual ramifications, and ramification size distributions. In addition, there are countless other attributes and distributions that can be easily computed from a QSM. A QSM consists of simple building blocks, which usually are some geometric primitives. For this particular case, circular cylinders are used being the most robust choice and a very accurate choice for estimating diameters, lengths, directions, angles, and volumes.

This modeling approach consists of, first, segmentation into ramifications, and second, fitting cylinders into these ramifications (Xu et al., 2018). The segmentation uses small surface patches to identify bifurcations along the root by computing the local connectivity of a moving region (Raumonen et al., 2013). This region-growing approach results in several connected non-bifurcated parts of the roots as segments. Notice that the segmentation starts from the base of a segment and then subdivides it into small successive pieces or layers. The segmentation process also reveals the topological root structure (relations of the child and parent for each ramification). After the segmentation, the segments are locally approximated as a sequence of cylinders with dimensions and orientations that could vary. Moreover, the succession relations of the fitted cylinders are also recorded (child/parent relation of the cylinders). When all the segments are reconstructed with cylinders, the cylinder model may still be refined. There may be small gaps between cylinders, so we fit cylinders to these gaps using only the previously fitted cylinders as data. Later, the cylinder model of the whole root is completed.

**FIGURE 1:**
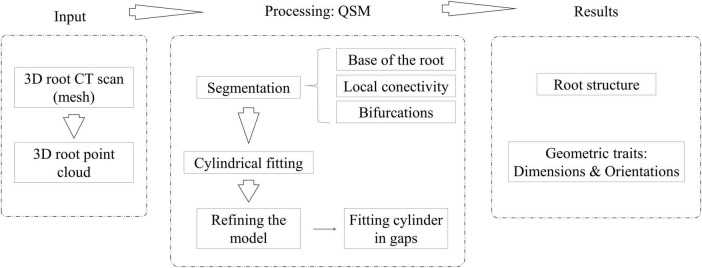
Workflow of 4DRoot, i.e., required input, the quantitative structure models (QSM) processing, and the computed results.

First, 3D CT scans in *stl* format as 3D meshes are the inputs. The mesh is transformed into a 3D point cloud by choosing the points where the curvature changes (OuYang and Feng, 2005). In addition, we have introduced a new variable called the *scale factor* (SF) that determines the tolerance between the fitted cylinder and the point cloud section that the model is approximating, directly affecting the minimum length of the fitted cylinders. This is the only parameter that has to be set up by the user to run 4DRoot. Next, several QSMs are generated due to random elements in the model reconstruction process. The variables of TreeQSM (Raumonen et al., 2013) are already optimized and fixed based on the possible dimension and resolution of the root scans. Through a statistical analysis, we have obtained the median model by studying the volume of the total root. Root traits, such as orientations and sizes of the main or lateral roots, and their size distribution are extracted from the QSMs, as well as the topological root structure. Figure 1 illustrates the workflow of 4DRoot, i.e., required inputs, the summarized processing, and the computed results.

### Registration

When temporal root scans are evaluated, the registration of these time-series data is required. The approach is based on the iterative closest point (ICP) algorithm (Ezra et al., 2008) which finds the affine transformation matrix that minimizes the distances between the closest points from overlapping areas of the two scans considered. The method handles the full six degrees of freedom by free-form curve matching (Besl and McKay, 1992). For this particular case, only a set of rotations is used. Moreover, the algorithm requires no extracted features, no curve or surface derivatives, and no preprocessing of 3D data, except for the removal of statistical outliers.

### Execution

To be able to execute the code, libraries from MATLAB such as “Computer vision toolbox,” “Partial differential equation,” and “Statistics and machine learning toolbox” have to be installed. The main path is where “rootQSM.m” is located. In addition, the MATLAB path of the subfolders where all the code of the software is has to be set within the MATLAB interface. The 3D root meshes in *stl* format coming from the X-ray CT system are the inputs. When “rootQSM.m” is run, the name of the file to process and the SF are requested by the console. 4DRoot also offers the possibility to view the 3D scan coded by depth (from yellow to blue) already scaled by the SF. Once “rootQSM.m” is executed, traits extracted from the QSM are numerically summarized in an excel file grouped into several sheets:

I *Total traits*: volume, depth, length, and area of the main and lateral roots, number and order of ramifications, root ball diameter, convex-hull and alpha-shape root ball area and volume (area of the planar projection’s convex hull and alpha shape of the root ball; volume of root ball’s alpha shape and convex hull), and 3D coordinates of the base of the root.
II *Ramification order traits*: volume, area, length, and number as a function of ramification order.
III *Taproot taper*: taproot taper function, where the first row is the distance along the main root and the second row is its diameter.
IV *Spread*: horizontal spread of the root in 18 directions and in 10 depth layers.
V *Cylinder distribution*: geometric characteristics of the fitted main root cylinders grouped into several classes:
  a. total volume, area, and length as a function of the diameter distribution of the cylinders (diameter classes by adding 0.1 mm per each class);
  b. total volume, area, and length as a function of the depth distribution of the cylinders (depth classes by adding 1 cm per each class);
  c. total volume, area, and length as a function of the zenith distribution of the cylinders (angle classes by adding 10° angle per each class);
  d. total volume, area, and length as a function of the azimuth distribution of the cylinders (angle classes by adding 10° angle per each class).
VI *Lateral root distribution*: geometric characteristics of the fitted lateral root cylinders grouped into several classes:
  a. lateral root volume, area, length, and number as a function of the diameter distribution of the lateral root cylinders (diameter classes by adding 0.1 mm per each class);
  b. lateral root volume, area, length, and number as a function of the depth distribution of the lateral root cylinders (depth classes by adding 1 cm per each class);
  c. lateral root volume, area, length, and number as a function of the azimuth distribution of the lateral root cylinders (angle classes by adding 10° angle per each class);
  d. lateral root volume, area, length, and number as a function of the distribution of the lateral root cylinders (angle classes by adding 10° angle per each class).

The units of the computed traits and geometric distribution determined on the excel sheet depend on the dimensionality of the trait, the units of the input CT scan, and the SF setup:

• [L] as length and depth: [*ud*]*fromtheCTscan*/*SF*
• [L^2^] as area: [*ud*^2^]*fromtheCTscan*/(*SF*^2^)
• [L^3^] as volume: [*ud*^3^]*fromtheCTscan*/^(*SF*^3^)*^10^3^

### Validation

The variability of the traits according to the SF setup by the user is statistically evaluated to estimate the robustness of 4DRoot. The computed traits might not follow a Gaussian distribution due to outliers. It means that statistics like the mean and the standard deviation will not provide a suitable analysis (Goodman, 1963). For this reason, the median and the normalized median absolute deviation (NMAD) (Eq. 1) are adopted as nonparametric and robust estimators. NMAD was employed as a substitute for standard deviation error where Gaussian distributions were not detected, while the central tendency was reported as the median. The NMAD allows comparing error dispersions from Gaussian samples, since it is normalized by the inverse of the cumulative distribution function of the Gaussian. Nevertheless, where deemed necessary by the normality of the dataset’s distribution, further mean and standard deviation calculations were also reported.


(1) N⁢M⁢A⁢D=1.4826*M⁢A⁢D

where *MAD* = *m*(|*x*_*i*_−*m*_*x*_|), the median (m) of the absolute deviations from the data’s median (m_*x*_).

## Experimental results

In this section, not only spatial but also temporal dynamics results by modeling 3D root scans were analyzed. For that purpose, black walnut tree scans in *stl* format served as input, a punctual scan for the spatial study and a weekly time-series scan for the temporal one. The pot where the root was planted was cylindrical with 180 mm diameter and 400 mm height. The growth medium was sifted sphagnum peat moss. The X-ray CT system manufacturer was Fraunhofer IIS (Fraunhofer Development Center X-ray Technology, Germany). The resulting cubic voxel size to scan was set at 100 μm^3^ where the minimum detectable root diameter was in the region of 0.5 mm. A RootForce tool (Gerth et al., 2021) was used for the segmentation process between root and soil.

**FIGURE 2:**
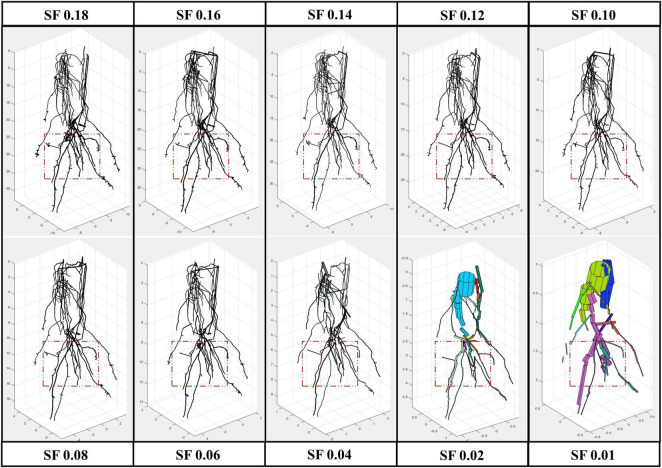
Root model results from the same scan using several scale factor (SF) values, from 0.01 to 0.18 with a remarked window in red where zoom is provided (Figure 3).

**FIGURE 3:**
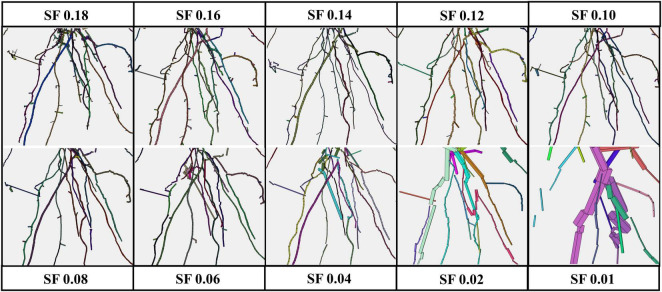
Zoom of the red window from Figure 2 [root model results using several scale factors (SFs) from 0.01 to 0.18].

All the experimental results obtained by running 4DRoot were run on a 2.5-GHz desktop computer with an Intel CORE I9 CPU and 32-GB RAM. First, spatial results from the same root scan using a range of SFs are shown. Notice that the only parameter that has to be set up for the user is the SF. The segmented 3D scan has 3.472.392 faces and 1.735.856 vertices. In Figure 2, the spatial-based modeling results are shown using 10 different SFs, namely, 0.18, 0.16, 0.14, 0.12, 0.10, 0.08, 0.06, 0.04, 0.02, and 0.01. Note that the SF determines the tolerance and, subsequently, the minimum length of the approximated flexible cylinders. A zoom window marked as a red rectangle in each model is recreated in Figure 3, where the root ramifications can be visualized in more detail. Figures 2, 3 clearly show how the results vary when SF changes. There is a wide range of SF values where the obtained models are very similar. Visually, it is easy to detect this range. When the SF is too small, the fitted cylinders are dimensionally too big.

In Table 1, several total traits already computed from the cylindrical model are quantified using different SFs. In this way, we are able to appreciate the variations for a set of values of the SF. The root traits are volume, depth, length, area, number of ramifications, root ball diameter, and convex-hull root ball area and volume.

**TABLE 1:** Total traits computed using different scale factors: volume, depth, length, area, number of ramifications, mean and maximum root ball diameter, convex-hull root ball area and volume, and 3 fractions between volume, area, and length.

Traits/SF	0.01	0.02	0.04	0.06	0.08	0.10	0.12	0.14	0.16	0.18
Volume (cm^3^)	48.34	4.60	1.29	1.02	1.13	1.22	1.11	1.05	1.17	1.14
Rooting depth (cm)	23.73	23.80	24.90	23.62	23.69	23.61	24.00	23.67	24.19	23.82
Length (cm)	138.6	247.8	326.9	370.5	358.4	392.9	414.3	411.1	407.8	463.2
No. of Ramif.	14	42	88	134	117	204	215	233	218	365
Area (cm^2^)	173.5	92.71	67.36	65.99	67.85	68.25	72.10	68.68	72.70	72.38
BallDAv (cm)	6.43	7.76	8.39	8.43	8.65	8.69	8.89	8.97	8.86	9.07
BallDMax (cm)	11.50	11.48	12.30	12.44	12.45	12.42	12.38	12.28	12.39	12.36
BallAreaCH (cm^2^)	83.84	84.19	89.14	89.94	89.89	89.62	92.00	91.18	92.54	92.70
BallVolCH (cm^3^)	0.10	0.09	0.08	0.10	0.10	0.10	0.10	0.10	0.09	0.10
%Volume/Area	27.86	4.96	1.91	1.55	1.67	1.79	1.54	1.53	1.61	1.58
%Volume/Length	34.87	1.86	0.39	0.28	0.32	0.31	0.27	0.26	0.29	0.25
%Area/Length	125.2	37.41	20.60	17.81	18.93	17.37	17.41	16.71	17.83	15.63

**FIGURE 4:**
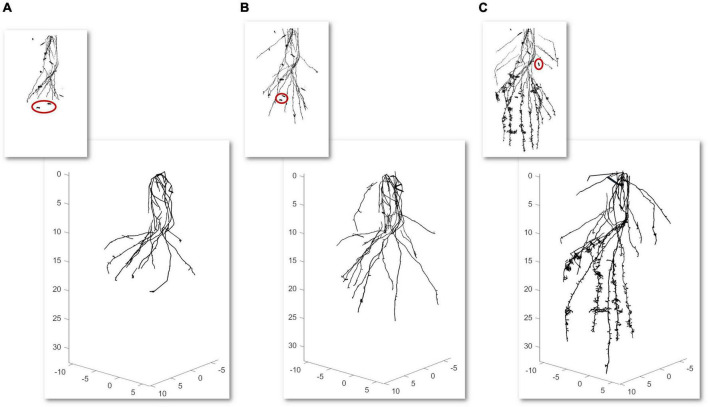
Time-lapse modeling of 3 weekly computed tomography (CT) scans scale factor (SF of 0.1) from the same root sample (axes in cm) **(A–C)** with a scan miniature in the upper part. Samples of noise reduction from the scans are marked in red.

Second, a temporal analysis from the same root is summarized. In this case, a weekly temporal scan is performed three times for the same root. We realized alignments between scans to be able to geometrically make a comparison between models. First, an approximate registration is manually done by picking similar pairs of points from the scan of the first week with the second week and this with the one from the third week. Next, the precise registration is carried out by the ICP algorithm using an overlap of 35% for the first case and 40% for the second case. Models from 3 weekly scans (axes in cm) with their CT scan miniatures in the upper part are illustrated in Figure 4. The segmented CT scans used as inputs have 2.387.896 faces (Figure 4A), 3.311.088 faces (Figure 4B), and 9.111.316 faces (Figure 4C). As shown in Figure 4, the majority of the noise in the CT scans (some samples marked in red), commonly provided by the segmentation between soil and root, is automatically removed in the modeling results.

Table 2 defines several total traits already computed from the cylindrical model for the 3 time slots. In that way, we are able to quantify and analyze the temporal variations.

**TABLE 2:** Total traits computed from a 3-week slot time: volume, depth, length, area, number of ramifications, mean and maximum root ball diameter, and convex-hull root ball area and volume, and 3 relations between volume, area, and length.

Traits/slot time	Week 1	Week 2	Week 3
Volume (cm^3^)	0.75	1.13	2.25
Rooting depth (cm)	19.29	25.17	32.41
Length (cm)	244.60	387.19	734.91
No. of Ramif.	104	135	671
Area (cm^2^)	44.90	66.12	132.53
BallDAv (cm)	8.71	11.18	14.66
BallDMax (cm)	13.63	17.82	18.00
BallAreaCH (cm^2^)	90.22	192.04	237.69
BallVolCH (cm^3^)	0.94	2.84	4.65
%Volume/Area	1.67	1.70	1.70
%Volume/Length	0.31	0.29	0.31
%Area/Length	18.35	17.08	18.03

## Discussion

In this section, we will start discussing the variability of the spatial results computed by using several SF values. According to Table 1, when the SF increases, the cylinder’s depth and radius are smaller for which the roots are better fitted (the tolerance is smaller), and the volume and area decrease. However, the cylinder’s size does not affect that much for detecting small branches because this is carried out during the segmentation process in the original TreeQSM (Raumonen et al., 2013). Nevertheless, by analyzing the root’s length, we can determine that it is directly proportional to the SF because the thin roots can be easily detected. This effect is due to the bigger dimensional size of the root because the rest of the parameters used for the segmentation process are fixed. In addition, different statistics are calculated in Table 3. For that case, the SF series chosen was 0.01, 0.02, 0.03, 0.04, 0.05, 0.06, 0.07, 0.08, 0.09, 0.10, 0.11, 0.12, 0.13, 0.14, 0.15, 0.17, and 0.18. Since the possible presence of outliers when using the values of SF is inadequate, the median and the NMAD serve as robust estimators. These are the cases when SF is 0.01 and 0.02. The greater the NMAD values, the further the data tend to be dispersed. This variation can be accepted for the majority of the traits due to the small values. For many traits, the results are very close to each other for a wide range of SFs, and thus the method can be concluded as robust from the point of view of a range of SF values. As mentioned earlier, length and number of ramifications are the traits with a larger NMAD because the SF affects the quantity of the detected roots. It is worth noting that when we consider a normal distribution at a 95% confidence interval (CI), the NMAD is more similar to the standard deviation (STD).

**TABLE 3:** Statistics of different traits calculated from an SF range from 0.01 to 0.18 [mean, standard deviation (STD), median, mean, and confidence interval (95% CI), STD (95% CI), median (95% CI), and normalized median absolute deviation (NMAD)].

Traits	Mean	STD	Median	Mean (95%)	STD (95%)	Median (95%)	NMAD
Volume (cm^3^)	1.62	11.08	1.18	1.17	0.10	1.14	0.17
Rooting depth (cm)	23.89	0.36	23.74	23.91	0.39	23.69	0.14
Length (cm)	353.74	75.98	381.71	390.67	34.44	400.08	38.10
No. of Ramif.	136.39	87.41	182.00	180.79	70.84	204.00	73.39
Area (cm^2^)	75.52	24.67	70.88	70.27	2.97	69.71	3.59
BallDiamAve (cm)	8.50	0.64	8.69	8.75	0.26	8.86	0.40
BallDiamMax (cm)	12.25	0.29	12.35	12.35	0.07	12.38	0.07
BallAreaCH (cm^2^)	89.86	2.55	90.29	90.63	1.53	90.50	1.51
BallVolumeCH (cm^3^)	0.30	0.62	0.10	0.32	0.62	0.10	0.02
%Volume/Area	2.14	6.16	1.67	1.66	0.13	1.61	0.18
%Volume/Length	0.46	8.13	0.31	0.30	0.04	0.29	0.06
%Area/Length	21.35	25.37	17.82	17.99	1.33	17.74	1.65

To sum up, after this quantitative analysis, we can affirm that there is a wide range of SF values where the obtained traits are very similar. Therefore, it is quite easy for a user to set up this variable. An accurate SF value depends on the tortuosity and heterogeneity of the root scan. However, a basic rule is to use the SF to get an input root between 10 and 30 linear units of depth.

**FIGURE 5:**
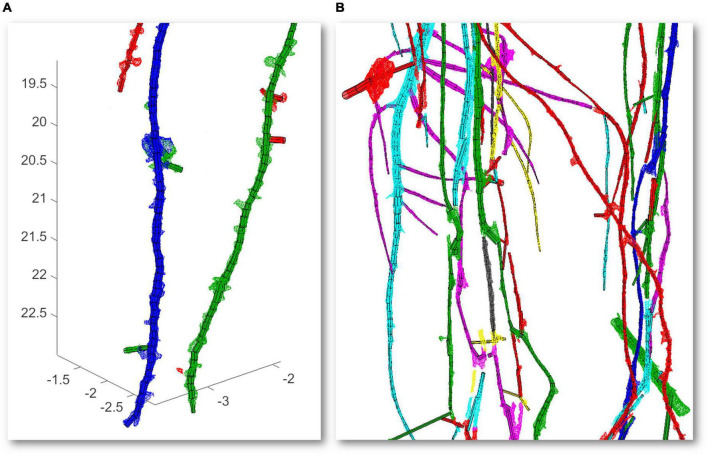
Overlapping between the cylindrical models represented with polyhedrons and the computed tomography (CT) scan scale factor (SF of 0.1) represented with dense points, both in colors depending on the ramification order: zoom with volume discrepancies detected lateral roots (axes in cm) **(A)** and zoom highlighted the complexity of the root **(B)**.

The volume of the digitally measured root is 1.855 cm^3^. It encloses all the noise coming from the ground-root segmentation process. The 4DRoot methodology is already validated with ground truth volume’s measurements using the digital root scan (Herrero-Huerta et al., 2021), while the focus of this study is to introduce the fully automatic 4DRoot software. Figure 5 shows the overlapping between the cylindrical model against the CT scan, colored depending on the ramification order, and how the lateral roots are detected. Figure 5A illustrates a zoom sample where the discrepancies on the volume can be discerned. Moreover, Figure 5B highlights the complexity of the root that 4DRoot has to face. These both samples show how the noise caused by the segmentation procedure between soil and root from the scan output is automatically removed in the cylindrical model.

**FIGURE 6:**
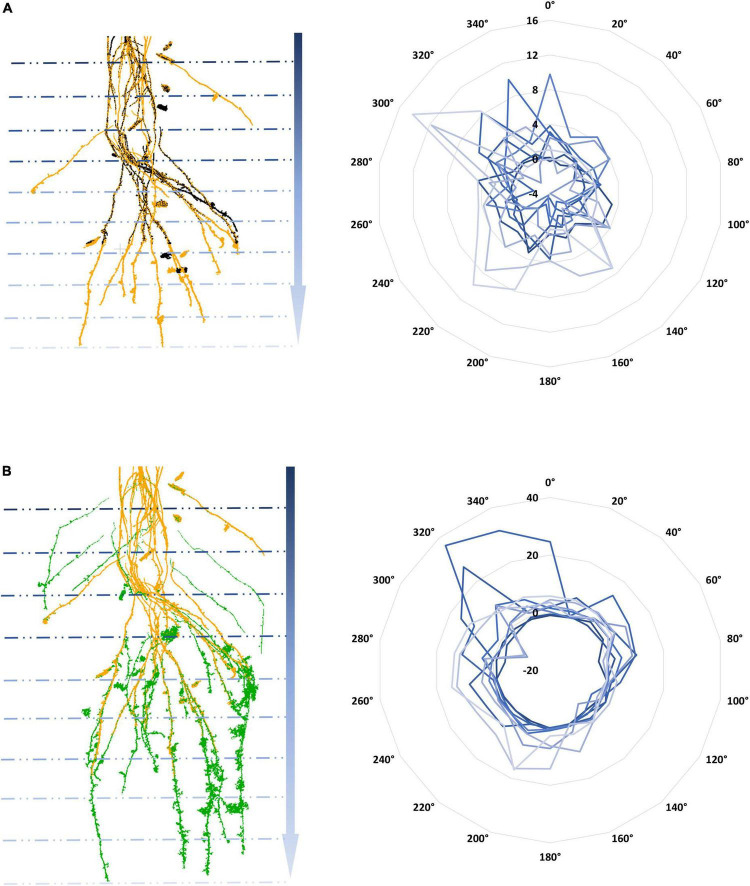
Temporal computed tomography (CT) scans overlap with a weekly time lapse: weeks 1 and 2 **(A)** and weeks 2 and 3 **(B)** in different colors on the left with its subsequent growth defined by the spread’s variations [computed by using an scale factor (SF) of 0.1] at 10 depths and 18 directions radially represented in blue scale color on the right.

Regarding the temporal dynamics of 4DRoot, Figure 6 illustrates the growth from 3 CT scans overlapping with a weekly time lapse defined by the spread’s variations at 10 equal depths and 18 directions, direct output from 4DRoot. This type of diagram easily shows the quantitative linear growth from 2 time-lapse scans in each direction along the depth. Figures 6A,B are in the same scale and orientation, being the yellow scan the same in both parts of the figure.

As a limitation, we affirm that ground truth volume’s measurements using the digital root scan show that the modeling reaches a smaller percentage error when parts of the root have a larger diameter (and vice versa). This is probably due to the increased relative uncertainty of the data for small roots. That is, even if the noise and other uncertainties in the data are evenly distributed over small and large roots, their effects on modeling smaller roots are bigger, hence, the higher percentage error for smaller roots. Another point is that 3D root scans usually have noise coming from the segmentation between soil and root which is still a semi-automatic process. As an advantage, the QSM can remove this noise because the 3D geometry of the roots is explicitly modeled.

## Conclusion

4DRoot tackles the extraction of several root phenotyping traits, making it a fully automatic, fast, accurate, and sufficiently robust way to process X-ray CT scans, with the outstanding advantage of the computationally low-cost requirement by removing the noise induced by the soil–root segmentation process and precisely detecting lateral roots. Furthermore, 4DRoot is able to analyze temporal 3D scans, evaluating both spatial and temporal dynamics of roots. The user gives the CT mesh scan in *stl* format as an input and receives the excel file with all the traits and geometric characterization as an output. As for interaction, the only parameter that has to be set up for the user is the SF.

4DRoot opens a large range of possibilities to provide scalability to a comprehensive analysis in order to advance high-throughput root phenotyping, with direct applicability in marker-assisted breeding and genetic mapping. Furthermore, the data generated quantify the contribution of structural root traits to crop development, supplying our understanding of the relationship between the plant phenome and plant function in ecosystems and efficiently improving nitrogen capture, water uptake, and carbon sequestration. All this information is vital to functional phenomics and potentially beneficial to combat major global challenges such as climate change, environmental degradation, and food insecurity (York, 2019).

As 3D models from X-ray CT become a standard data type to noninvasively digitize RSA in lab conditions, we envision that 4DRoot will contribute to moving the next generation of root phenotyping forward.

## Data availability statement

The original contributions presented in this study are included in the article and at https://github.com/TIDOP-USAL/4DRoot, further inquiries can be directed to the corresponding author MH-H, monicaherrero@usal.es.

## Author contributions

MH-H conceived the idea, developed the data analysis pipelines and software, performed the data analysis and visualization, and wrote the manuscript. DG-A and PR supervised the research and edited the manuscript. All authors read and agreed to the published version of the manuscript.

## Footnotes

1
https://github.com/InverseTampere/TreeQSM
